# Density Functional Theory Investigation of 2D Phase Separated Graphene/Hexagonal Boron Nitride Monolayers; Band Gap, Band Edge Positions, and Photo Activity

**DOI:** 10.1021/acs.jpcc.4c06121

**Published:** 2024-11-27

**Authors:** Eoin M. O’Sullivan, Nicole Grobert, Marcel Swart

**Affiliations:** † Department of Materials, University of Oxford, Oxford OX1 3PH U.K.; ‡ Institute de Quimica Computacional i Catálisi, Universitat de Girona, Girona 17003 Spain; § ICREA, Pg., Lluís Companys 23, Barcelona 08010, Spain

## Abstract

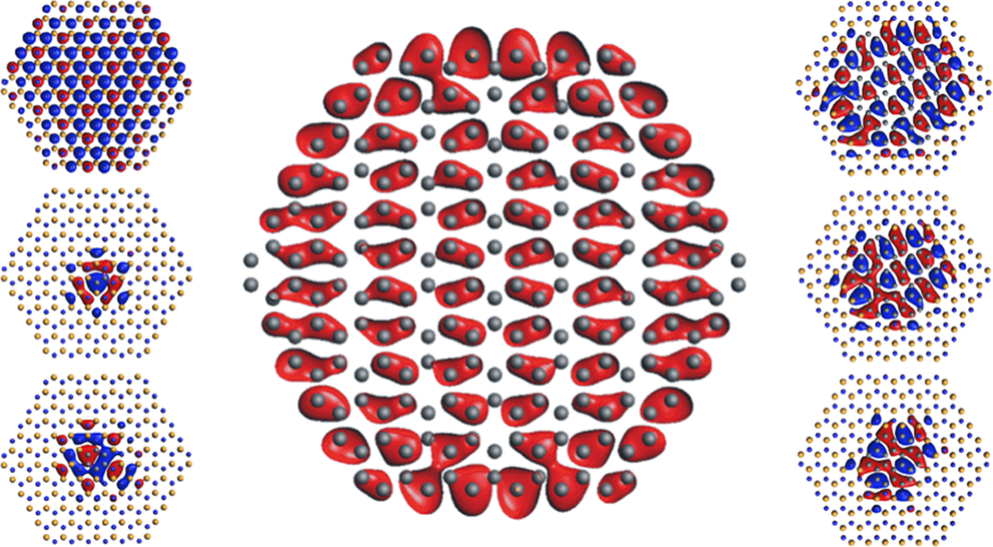

Creating sustainable and stable semiconductors for energy conversion via catalysis, such as water splitting and carbon dioxide reduction, is a major challenge in modern materials chemistry, propelled by the limited and dwindling reserves of platinum group metals. Two-dimensional hexagonal borocarbonitride (h-BCN) is a metal-free alternative and ternary semiconductor, possessing tunable electronic properties between that of hexagonal boron nitride (h-BN) and graphene, and has attracted significant attention as a nonmetallic catalyst for a host of technologically relevant chemical reactions. Herein, we use density functional theory to investigate the stability and optoelectronic properties of phase-separated monolayer h-BCN structures, varying carbon concentration and domain size. We find that, on average, a higher C content reduces the energetic cost of carbon inclusion per atom, as an increasingly graphitized network lowers the overall energy of the structure. Using functional HSE06, we show how the electronic bandgap of h-BN can be reduced from 5.94 to 1.61 eV with significant substitution of C in the domain (C at. % ∼ 44%) adding to the weight of evidence that suggests these segregated h-BCN systems can easily be customized. We use the location of conduction and valence band edges with respect to the potentials of HER, OER and CO_2_ reduction to assess the catalytic suitability of these materials, identifying three structures with appropriate band edges for these catalytic reactions. Finally, the photoactivity of the structures is assessed through TD-DFT calculations, and we propose two candidates for photocatalysis based on the segregated h-BCN system.

## Introduction

Low-dimensional materials have attracted continuous interest in material science fields where they are employed as functional building blocks. These building blocks can be used to create highly efficient macrostructures that benefit from the unique properties of these functional materials and therefore can offer cost-effective replacements for traditional materials which rely on precious resources. Hexagonal borocarbonitrides (h-BCN), part of the 2D materials family and a class of metal-free heterogeneous semiconductors, have emerged as environmentally friendly and highly selective catalysts for a variety of reactions.^1−11^ Often considered as a chemical mixture of hBN and graphene, h-BCN materials have exceptional properties, complementary to those of pure hexagonal boron nitride and sp^2^ carbons, such as varied surface functional groups and electronic structure.^12,13^ As such, h-BCN materials have exhibited notable activity enhancement in catalytic tests against their carbon or boron nitride counterparts^6,9,14^ as well as industry standards.^2,8^ However, h-BCN as a catalytic material is still at an early stage of its development, with the relationship between atomic design and catalytic activity of the materials yet to be fully understood. This partly due to the wide variety of bonding iterations the material can have as well as the lack of practical knowledge regarding the distribution of B, C, and N within synthesized h-BCN materials. This wide range of permutations within the h-BCN network also imparts a high degree of tunability with regards the optical and electronic properties of the material, based on two factors: the atomic proportion of B, C, and N in the structure and, second, the distribution of atoms throughout, giving various possibilities of B–C, B–N, C–C, and N–C bond formation. This adaptability has seen h-BCN materials engineered in a wide variety of applications from electrochemical sensing, catalysis, and energy storage.^2,4−,6,15−22^

As such, in order to understand the relationship between the structure of h-BCN and its properties better, theoretical investigations have focused on the atomic arrangement within the material, often exploring highly entropic and exotic “homogeneous” h-BCN systems which offer exciting properties, such as ultralow bandgap,^23−28^ but are difficult to realize experimentally. The chemical stability of h-BCN depends on the relative energy of the bonding interactions between B, C, and N atoms in the structure. Considering system thermodynamics, h-BCN materials tend to form B–N and C–C bonds over B–C/N–C bonds as they are energetically more stable. Looking at bond energies, which is a key factor in stability, the trend is as follows; B–N (4.00 eV) > C–C (3.71 eV) > N–C (2.83 eV) > B–C (2.59 eV).^29^ By maximizing the number of B–N and C–C bonds, the overall structure is increasingly more stable.^30,31^ Evidently, this theoretical observation is also found experimentally, with most h-BCN synthesis studies concluding that the materials synthesized are comprised of segregated h-BN and graphene nanodomains.^32−34^

**Figure 1:**
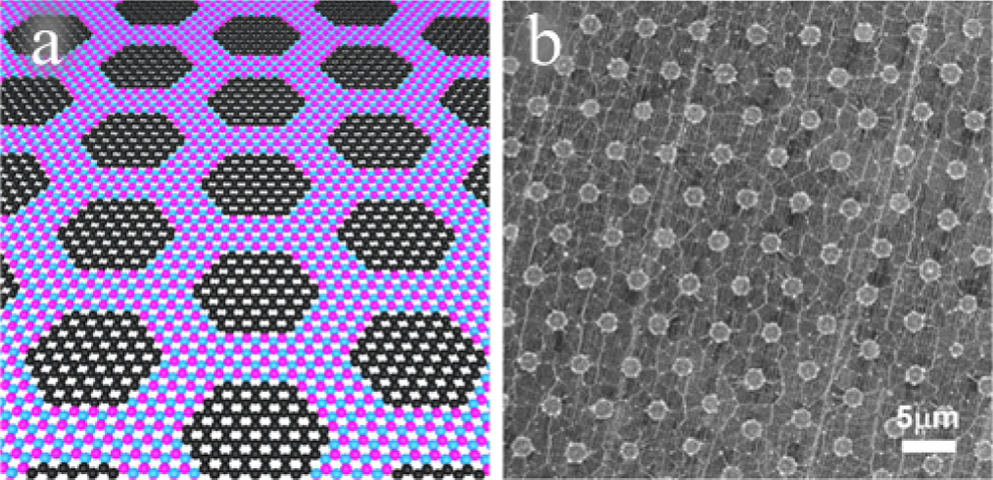
(a) Schematic and (b) scanning electron microscopy image of the superordered arrays of phase-separated h-BCN synthesized by Li et al.,^35^ showing how ordered h-BCN structures with tailorable h-BN and C domain sizes are possible at the macroscale. Reproduced from.^35^ Copyright 2022, American Chemical Society.

As graphene/h-BN segregation is almost unavoidable, much effort has instead been focused on controlling relative domain sizes and concentration in the films. Recent efforts in synthesizing h-BCN materials have resulted in centimeter scale, h-BN-graphene superordered arrays comprised of graphene domains in a h-BN matrix.^35,36^ In particular, M. Li et al. demonstrated precise control over the domain sizes of h-BN:C and their density, through simple operating parameters such as the CH_4_:H_2_ flow ratios and temperature of the growth substrate (Figure 1).^35^

With such control over the domain sizes and array densities in these h-BCN structures, it will soon be possible to create h-BCN films with defined C:h-BN domain sizes, such that structures with specific optical and electronic properties can be realized, tailored toward the specific applications. To guide this work, a clearer understanding of the fundamental electronic properties of the composite structures is useful. More specifically, an understanding of the effects of the interface between domains and their relative sizes on the optoelectronic parameters can inform system design in these quasi-crystalline arrays, in order to identify trends and earmark potential structures suitable for further study.

In the present work, we investigate the optoelectronic properties of symmetrically similar, phase-separated structures of h-BCN to those recently reported.^35^ We use the finite flake model to analyze structures of 252 atoms and employ first-principle calculations to systematically explore their optoelectronic properties under gradients of carbon concentration and domain size. Based on this, we identify viable structures that show potential as photoactive, catalytic materials for use in the Hydrogen Evolution Reaction (HER), Oxygen Evolution Reaction (OER) and the methanation of CO_2_.

### Computational Details

**Figure 2:**
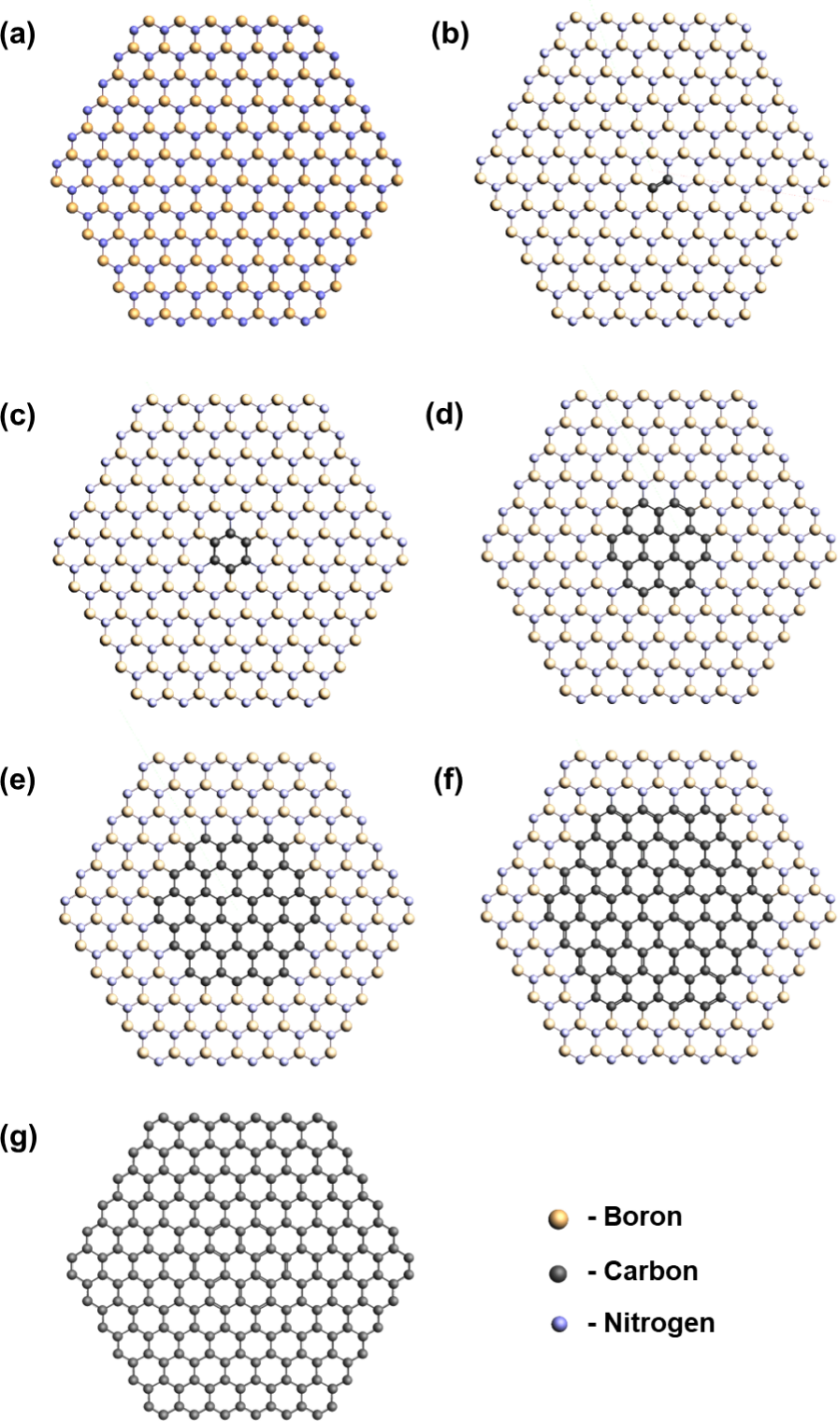
Atomic structures of calculated monolayer BN-C islands with C concentrations (a) 0%, pure BN, (b) 0.9%, BN-C2 (c) 2.8%, BN-C6, (d) 11.1%, BN-C24, (e) 25.0%, BN-C54, (f) 44.4%, BN-C96 and (g) 100%, Gr. Hydrogen edge atoms are not shown but are part of the calculated structure.

Density Functional Theory (DFT) calculations were performed via the Amsterdam Modeling Suite (AMS) licensed by Software for Chemistry and Materials. Geometric optimizations of the structures were performed with the BP86 functional with D3 dispersion correction and all electron STO ZORA – DZP basis set. We explore various h-BCN structures that are constructed by doping the center of a hexagonal h-BN domain. The initial carbon concentration is 0.9% (BN-C_2_ - C dimer), 2.8% (BN-C_6_ - six C ring) and then gradually increasing the size of the hexagonal C domain until a pure graphene flake is realized. All edge atoms of the modeled systems are passivated with hydrogen atoms, ensuring that all atoms are fully coordinated. Figure 2 shows each of the systems investigated in this study.

To evaluate the energetic stability of these BCN structures with respect to the intrinsic h-BN structure, their formation energies (E_form_) were calculated. E_form_ is an important parameter that helps to estimate whether a substitution is feasible or not and it is defined as
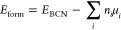
1where E_BCN_ is the total energy of the BCN monolayer, E_BN_ the total energy of a carbon-free domain, n_i_ is the number of atoms for each element (i = B,N,C), and μ_i_ is the corresponding chemical potential. The chemical potentials μ_B_, μ_N_, and μ_C_ must meet the conditions of thermodynamic equilibrium, such that
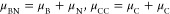
2where the parameters μ_BN_ and μ_CC_, are the chemical potentials for the boron–nitrogen (BN) and carbon–carbon (CC) pairs, respectively. In this study, the chemical potentials for the CC and BN pairs were derived by taking the BN flake and graphene flake as references and assigning zero values to their formation energies i.e., μ(BN) = E_BN_/n_BN_ and μ(CC) = E_Gr_/2n_C_. Since n_B_*=* n_N_*=* n_BN_ and n_CC_*=* n_C_/2, for all cases, eq 1 can be written by using eq 2 as
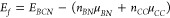
3

For electronic structure calculations, several XC functionals were tested for increased accuracy against BP86-D3.^37,38^ These include the revised TPSS functional,^39,40^ Tran-Blaha modified Becke-Johnson (TB-mBJ),^41,42^ and the Heyd, Scuzeria, and Ernzerhof hybrid functional (HSE06 - within the Amsterdam Modeling Suite (AMS) software, the switching parameter, ω, was set at 0.11),^43,44^ the results of which can be found in the(Table S1). To determine which functional was the most accurate, we compared bandgap values obtained for the intrinsic h-BN case with the experimental bandgap of monolayer h-BN at 6.05 eV.^45,46^ The band edge potentials for the systems modeled were also compared against literature values for the band edge positions of h-BN with respect to vacuum (Table S2). It was found that the HSE06 XC functional could accurately model the optoelectronic properties and band edge positions of the system, which is essential for reliably assessing the photocatalytic potential in hydrogen evolution reaction (HER) and oxygen reduction reaction (ORR) applications.

In this study, we used a finite cluster model rather than a fully periodic model for representing the phase-separated h-BCN structures due to computational constraints when employing the Heyd-Scuseria-Ernzerhof hybrid functional. The selected cluster model allows us to simulate the core unit cell of the phase-separated structures observed in the study by Li et al.—a hexagonal carbon domain within a hexagonal h-BN matrix.^35^ This approach enables a balance between accuracy and feasibility, as it provides reliable electronic property trends over varying carbon concentrations and domain sizes without incurring the prohibitive computational expense of a large periodic model. In contrast, prior work employing periodic models and the HSE06 functional has been limited to relatively small systems (periodic cells of 18 atoms with up to 33% carbon doping, i.e., Six C atoms^23^ which are computationally manageable but do not represent the larger graphene and h-BN domains found in experimental phase-separated h-BCN arrays. Using a cluster model enables us to apply computationally demanding functionals such as HSE06 on a larger atom basis within a finite system, yielding accurate electronic properties for the structure, which are often underestimated by generalized gradient approximation (GGA) functionals such as PBE in large periodic models.^47,48^ While our model does not capture the extensive phase-separated arrays synthesized by Li et al., it most effectively represents the carbon domain variations of the basic unit cell, within the computational constraints of density functional theory, and provides valuable insight into the key electronic trends and properties that would likely be observed in an extended system.

## Results and Discussion

### Optimized Structures and Their Stability

The geometry optimized model systems are those shown in Figure 2. Table 1 shows the composition, band gap (E_g_) and formation energy (eq 3) per C atom added for each of the systems in this study.

**Table 1:** % C Composition, Formation Energy per C Atom and Band Gap Values for the BN-C Structures Analyzed in This Study

Structure	C at %	Formation Energy per C (eV/C)	Band Gap (eV)
BN	0.00		5.94
BN-C_2_	0.9	1.07	4.70
BN-C_6_	2.8	0.50	4.67
BN-C_24_	11.1	–0.17	3.04
BN-C_54_	25.0	0.21	2.15
BN-C_96_	44.4	0.17	1.61
Gr	100		0.95

**Figure 3:**
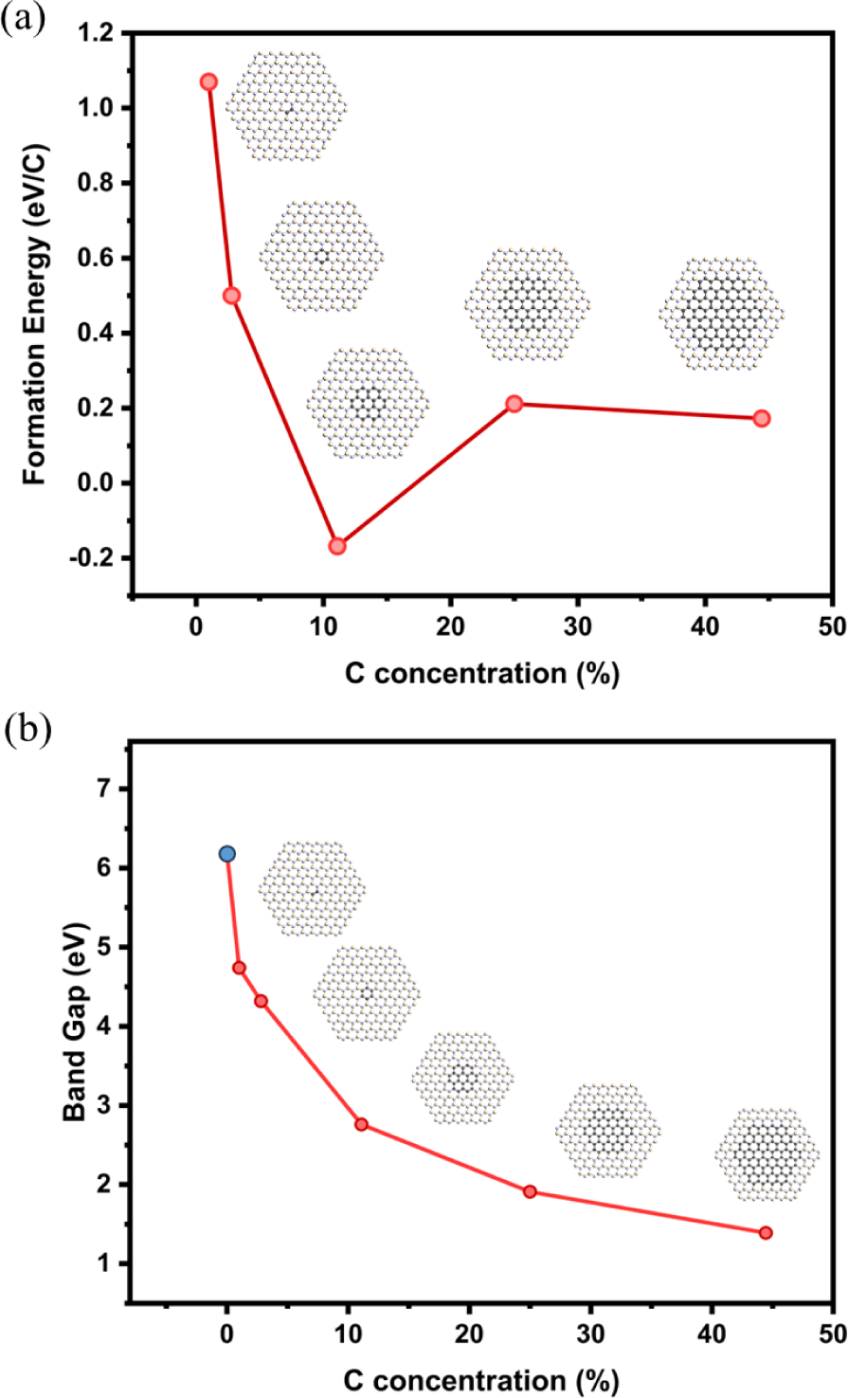
(a) Formation energies per C atom introduced vs the carbon concentration of the structure. (b) Electronic band gap vs carbon concentration of each structure. Blue point is the intrinsic h-BN case.

Figure 3a shows the trend in formation energies per C atoms. Disrupting the symmetry of the h-BN lattice comes with a high energetic cost and we observe larger formation energies per C atom in the system for systems with low C atomic %. In the case of the C dimer, the formation energy is 1.07 eV/C atom which drops to 0.50 eV/C atom with BN-C_6_, as the π-conjugation in the C ring increases stability. The calculated formation energies per C atom match closely with those reported on BN_C2_ and BN_C6_ structures in DFT studies on C doped h-BN, validating our observed results.^49^ For BN-C_24_, the formation energy reaches a minimum and is negative (i.e., structure is more stable than intrinsic h-BN sheet). At higher C atomic %, the formation energy is positive once more. This result suggests that there is an optimal structure that shows increased stability which occurs when C content = 11%, i.e., where the ratio of the h-BN:C domain diameters is 3.6:1. Structures with larger carbon domains require an energetic cost, however, this value plateaus around 0.2 eV.

Evident in Figure 3b, the structures show a tunable band gap, ranging from 4.74 eV at lowest C inclusion (1%), down to as low as 1.61 eV with highest C at. % (44%), proving the viability of these segregated h-BCN systems as a tailorable electronic system. A sharp decrease in E_g_ is observed upon first C inclusion as the symmetry of the BN lattice is broken. Upon further C introduction and enlargement of the C domain, the bandgap decreases gradually to 0.95 eV for a pure carbon domain, i.e., the pure carbon flake is not gapless as expected for a graphene (Gr) sheet.^50^ This bandgap observed in such carbon flakes is due to its finite size, i.e., it is not a repeating structure with C–C terminated edges. Previous reports on hydrogenated graphene (e.g., graphane/graphone) have shown how the presence of C–H bonds in the structure can open up the band gap.^51,52^ Wei Hu et al. reported on the band structure of similar hexagonal, hydrogen terminated flakes of graphene and found that extended systems with diameters >6.7 nm (ca. 1500 C atoms) were required for a gapless band structure.^53^ As our system involves a finite flake size of diameter ∼2.4 nm with hydrogen passivated edges, to be able to accommodate the Gr-h-BN heterostructure, the HSE06 functional here predicts a nonzero bandgap for the intrinsic carbon case.

Comparing experimental data for the electronic bandgap of similar h-BCN structures produced by Li et al.,^35^ determined via STM measurements, structures with C content of around 18 at. % C (h-BN: C domain diameter ratio of 2.7:1) are determined to have a bandgap (E_g_) as 1.3 eV. This value for E_g_ lies below those calculated for structures with similar at. % C here (∼2 eV). One possible explanation for the deviation between experiment and theory could be the experimental details of the STM method, which have not been reported, mainly related to the substrate on which the film rests–copper (its growth substrate) or silicon dioxide. Such a parameter would significantly affect the electronic properties of the layer and result in a reading of E_g_ different to that determined here in vacuum. For example, Nicholas Lanzillo et al. previously reported the band gap renormalization of carbon nanotubes to smaller values on an insulating h-BN substrate from first principle calculations.^54^ The decrease in band gap is the result of a polarization-induced screening effect from the substrate which, in this case, altered the band gap by up to 0.5 eV. Other experimental data gathered on BCN films, comprised of randomly distributed h-BN:graphene nanodomains, showed that at 65 at. % C, the optical band gap was 1.62 eV as determined via UV–vis spectroscopy on optical quartz substrates, much closer to results gathered here for similar structures.^32^ In any case, further experimental references of band gaps in h-BCN materials are needed to confirm the accuracy of the HSE06 functional in this system.

**Figure 4:**
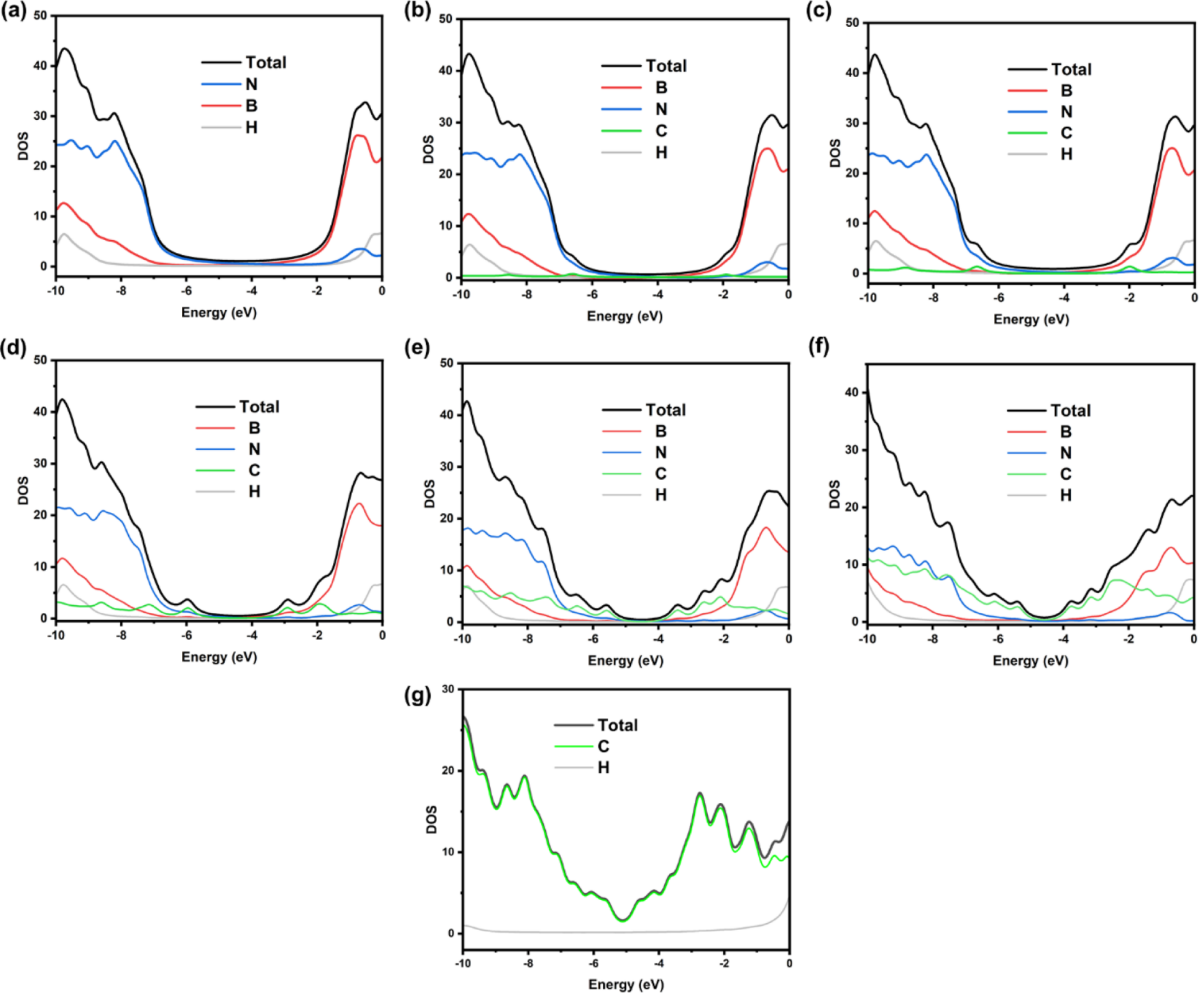
Partial density of states (PDOS) showing the atomic contributions of B, C, N and H in the (a) BN, (b) BN-C_2_ (c) BN-C_6_,(d) BN-C_24_, (e) BN-C_54_, (f) BN-C_96_ and (g) Gr systems.

The partial density of states showing the atomic contributions of B, C, N and H for the various structures are shown in Figure 4. For all C containing structures, we see that it is the C and N orbitals that contribute to the valence band levels whereas the conduction band levels are comprised mostly of B and C orbitals. States near the Fermi Level are composed solely of p orbital contributions (Figure S1). It is clear from the depicted results that the band gap of the structures is reduced with increasing size of the C domain. Moreover, further electronic states are added to the conduction band upon their addition (essentially, increasing the number of carbon atoms and, therefore, size of the carbon domain increases the density of electronic states close to the band gap). This reduction in the band gap will increase the electrical conductivity of the material, acquiring a semiconductor character.

**Figure 5:**
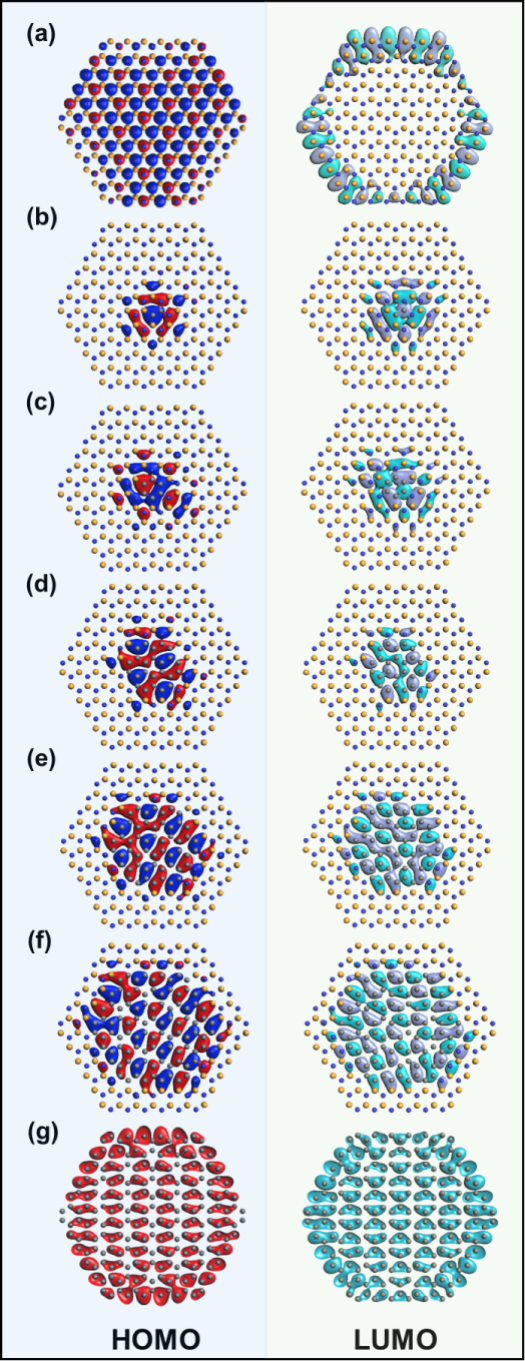
Highest occupied molecular orbital (HOMO) and lowest unoccupied molecular orbital (LUMO) for the various systems; (a) intrinsic BN, (b) BN-C_2_ (c) BN-C_6_ (d) BN-C_24_ (e) BN-C_54_, (f) BN-C_96_ and (g) Gr. C bonding states are the dominant feature of the HOMO and LUMO of each structure. Red and blue orbitals are opposite in phase in HOMO, similarly for green and gray in LUMO.

The influence of C on the electronic states of these materials is further illustrated in the electron distribution depictions of the highest occupied molecular orbital (HOMO) and lowest unoccupied molecular orbital (LUMO) for each structure (Figure 5). In the BN model, the HOMO is localized on each of the N atoms with no interaction with neighboring B atoms. The LUMO is comprised of the outermost B atoms. As C is introduced the HOMO and LUMO are located at the C domain and charge transfer only occurs via the interior carbon domain and domain boundary. Notably, in-phase interactions occur between C and B atoms. Here, orbitals exhibit mixing and are no longer localized as in the case of BN, suggesting electronic exchange between C and B atoms along the domain boundary. On the other hand, little charge transfer occurs between C and N atoms at this boundary point. However, the C domain does induce delocalization of electron density between B and N atoms in regions of h-BN near the domain interface, something not observed in the intrinsic h-BN case. In the pure carbon system, HOMO and LUMO orbitals reflect the delocalized nature of the graphene system as all orbitals are in phase with each other.

### Band Edge Positions and Alignment—Catalytic Suitability

To investigate the catalytic suitability of these h-BCN structures for applications in technologically important reactions such as HER/ORR and CO_2_ methanation, one must look at the band edge potentials for the materials at hand. To be suitable for redox catalysis, the material must satisfy the following thermodynamic conditions:

1. The HOMO is more positive than the oxidation potential of H_2_O (*E*° = 1.23 V vs NHE)^55^
2. The LUMO is more negative than the reduction potential of H^+^ (*E*° = 0.0 V vs NHE)^55^ or CO_2_ (E_CH4_° = −0.24 V vs NHE^56^

**Figure 6:**
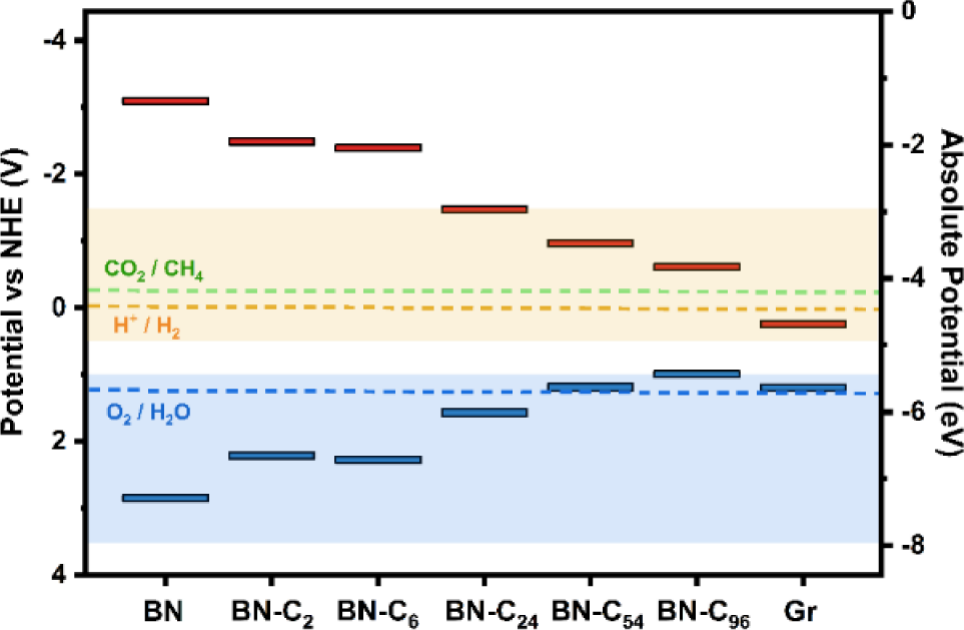
HOMO (blue) and LUMO (red) levels, i.e., band edges, of the BNC_*x*_ structures analyzed in this work in comparison to optimal band edge positions for HER/OER and CO_2_RR. The left *Y*-axis of the plot shows the potential difference with respect to Normal Hydrogen Electrode (NHE) potential, and the right *Y*-axis refers to the absolute energy with respect to vacuum. The potentials for HER (H^+^/H_2_) at 0 V vs NHE, OER (O_2_/H_2_O) at 1.23 V vs NHE and CO_2_RR (CO_2_/CH_4_) at −0.24 V vs NHE are depicted by orange, blue and green horizontal dashed lines, respectively and the optimal ranges for the materials band edges are shown by the similarly shaded regions.

Figure 6 shows how the band edge potentials of the BN domain are modified by the inclusion of carbon in the lattice. Here, the left *Y*-axis signifies the potential difference with respect to Normal Hydrogen Electrode (NHE) potential, and the right *Y*-axis refers to the absolute energy with respect to vacuum. The relative positions of the band edges with respect to the NHE is calculated using the standard redox potential with respect to the vacuum potential, which is taken as −4.44 V for hydrogen reduction.^55^ The potentials for hydrogen reduction reaction (H^+^/H_2_) at 0 V vs NHE and Oxygen Evolution Reaction (O_2_/H_2_O) at 1.23 V vs NHE are depicted by orange and blue horizontal dashed lines, respectively, and the optimal ranges for the materials band edges are shown by the similarly shaded regions. Hoffmann et al. previously reported that for optimal catalytic performance, the material should have its conduction bands at a chemical potential of +0.5 to −1.5 V vs NHE and its valence bands at a chemical potential of +1.0 to +3.5 V vs NHE^57^ and these intervals form the shaded regions in Figure 2. Furthermore, the green dashed line references the potential required for CO_2_ methanation at −0.24 V vs NHE.^56^ This can be extended to several other carbon species, depending on the reduction potential (HCOOH; −0.61 V, CO; −0.53 V, CH_3_OH; −0.38 V, C_2_H_4_; −0.35 V, C_2_H_6_; −0.31 V).^56^

**Figure 7:**
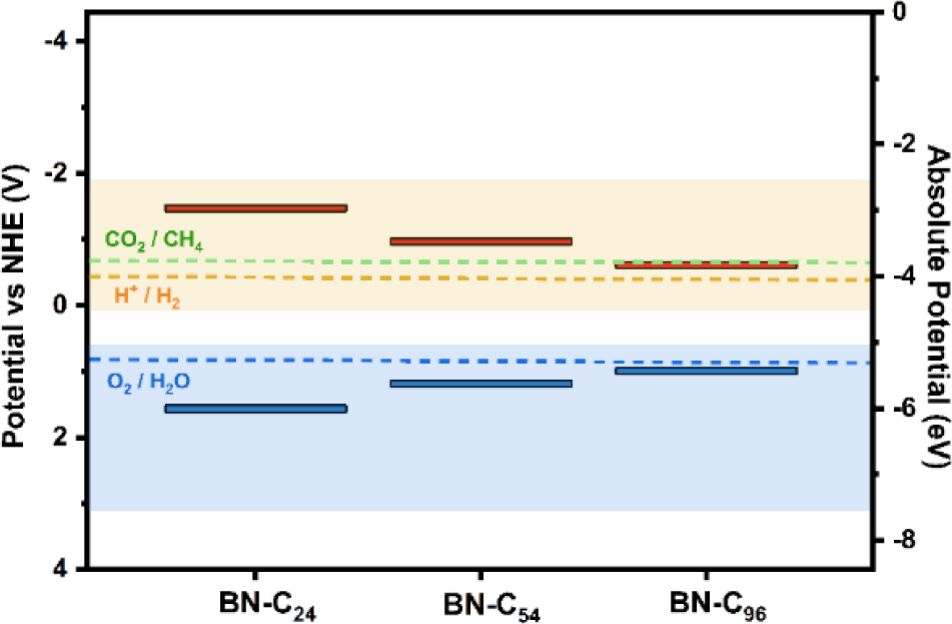
HOMO (blue) and LUMO (red) levels of the three optimal BN-C structures, BN-C24, BN-C54 and BN-C96, with the chemical potentials of HER, OER and CO2 reduction shifted by 0.41 V when pH = 7.

Table 2 shows the HOMO, LUMO positions and offset potentials for each h-BCN system investigated. In our calculated system, for intrinsic BN, the HOMO level already lies within the range for optimal oxidation energetics, as shown by the shaded blue region. However, with a band gap of almost 6 eV, the LUMO level is significantly higher than the optimal reduction energies, making it an unsuitable choice material for catalytic reduction. As carbon is introduced to the lattice, there is an expected narrowing of the band gap as both the HOMO and LUMO levels move toward each other. This trend continues even at the highest levels of carbon introduced. While BN-C_2_ and BN-C_6_ both satisfy the requirements for oxidizing performance, their LUMO still lie outside the chemical potential interval for optimal reduction of H_2_ and CO_2_. This suggests that slightly doped BN, such as that irradiated with ions for C implantation or MOVPE grown samples,^58−60^ may not be suitable for catalysis in the HER/ORR. With BN-C_24_, both requirements are satisfied as the LUMO enters the region for optimal reducing performance, particularly for oxidation of H_2_O, as the HOMO approaches the theoretical potential for the OER at 1.23 V. Still, there is a significant reducing offset of about 1.5 V. In the case of BN-C_54_, we find that the position of the HOMO is at 1.15 V vs NHE, which is just below that of the oxygen reduction requirements, and the LUMO is located at a more reasonable reducing offset of −1.01 V. Finally, with BN-C_96_, the highest C at. % system, the LUMO is at −0.66 V vs NHE, providing a reducing offset of 0.66 V for H_2_ and 0.42 V for CO_2_. The HOMO is located at a lower value of 0.95 V vs NHE and is therefore slightly above the optimal range. Despite good reducing performance, the absence of an oxidation offset here reduces the applicability of BN-C_96_. The band edge positions for the graphene flake are also included. Both HOMO and LUMO levels lie outside the optimal range to provide a good oxidation and reduction offset for the OER and HER, respectively. Furthermore, the band gap of the system (0.95 eV), the minimum energy of the photon harvested by the material, is below that required to drive OER (1.23 V). As such, the hydrogenated carbon nanoflake is not thought to be a good candidate for OER/HER catalysis. For systems BN-C_24_, BN-C_54_ and BN-C_96_, the band gaps are suitable. However, their band positions are not ideal. In order to improve upon this, parameters such as pH and temperature of the reaction can be varied in order to alter the potentials required.^23^

**Table 2:** HOMO, LUMO Levels for the Various h-BCN Systems Investigated, Their Chemical Potential vs NHE as well as Voltage Offset from Their Respective Oxidation and Reduction Potentials

System	HOMO (eV)	V vs NHE (V)	Oxidation Offset (V)	LUMO (eV)	V vs NHE (V)	H_2_ Reduction Offset (V)	CO_2_ Reduction Offset (V)
BN	–7.23	2.79	1.56	–1.3	–3.14	–3.14	–2.90
BN-C_2_	–6.60	2.16	0.93	–1.91	–2.53	–2.53	–2.29
BN-C_6_	–6.66	2.22	0.99	–2.00	–2.44	–2.44	–2.20
BN-C_24_	–5.96	1.52	0.29	–2.94	–1.50	–1.50	–1.26
BN-C_54_	–5.58	1.14	–0.09	–3.42	–1.02	–1.02	–0.78
BN-C_96_	–5.39	0.95	–0.28	–3.78	–0.66	–0.66	–0.42
Gr	–5.59	1.15	–0.08	–4.64	+0.20	+0.20	+0.44

At T and pH > 0, energy levels are shifted by (*k*_*B*_*T* ln10) x pH, where k_B_ is Boltzmann’s constant.^61^ At room temperature (298 K) and pH 7, this provides a shift of 0.41 V. Other studies have focused on engineering strain in the lattice as a method of shifting band edge positions into the desired range.^26 −28^ Under conditions of room temperature and neutral pH, Figure 7 shows how the HOMO and LUMO levels of the three optimal structures, BN-C_24_, BN-C_54_ and BN-C_96_ shift in energy level. Here, all three structures satisfy the thermodynamic requirements for HER and OER. BN-C_24_ is now below the oxidation potential with an offset of 0.70 V, while its LUMO lies 1.09 and 0.85 V above the reduction potential for H_2_ and CO_2_, respectively. For BN-C_54_, the HOMO is very well situated at 0.32 V below the potential for H_2_O. Similarly, its LUMO has a reduction offset of 0.61 V for H_2_ and 0.37 V for CO_2_, potentially making this a material with good reducing and oxidation capabilities. With BN-C_96_, we find that the position of the HOMO band now lies 0.13 V below the oxidation potential for H_2_O, giving it an oxidation offset and improving its applicability in the oxygen reduction reaction. The LUMO now has a reducing offset of 0.25 V for H_2_ and 0.01 V for CO_2_, making it an attractive candidate for HER although its suitability for CO_2_ reduction might be diminished as a small offset is generally required.^62^ Electrolysis in neutral pH presents a transformative way for environmentally friendly, cost-effective hydrogen production. However, the field is still developing with one of the biggest challenges being the lack of robust hydrogen evolution reaction (HER) catalysts that are active at pH 7.^63,64^ Here, we have identified three candidate systems which show good bifunctionality in the overall water splitting reaction at RT and neutral pH.

### Optical Propertiess—Oscillator Strength and Absorption

**Figure 8:**
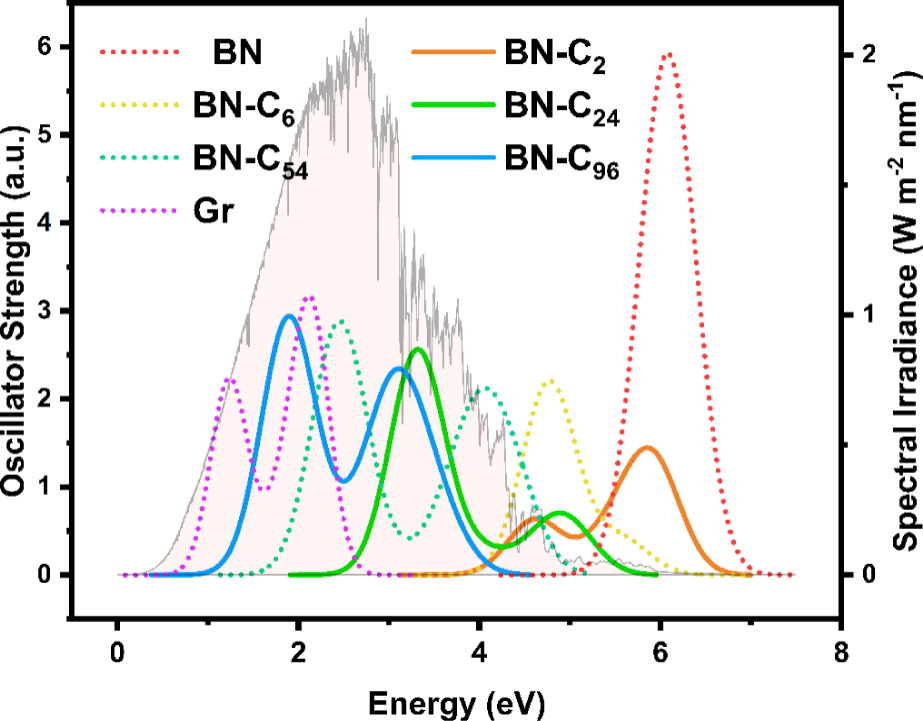
Oscillator strength with respect to excitation energy for the various BN-C structures showing their optical absorption properties. The solar spectral irradiance as a function of energy is watermarked in light orange to show the “photoactive” regions (quantified by the right-hand side *y*-axis).

Light absorption is an important factor if these materials were to be used in photocatalysis. The oscillator strength of each systems excitations is calculated, a quantity that can be directly related to light absorption.^65^ From this, we can infer the photoactivity of the material and its suitability in photocatalytic applications. Figure 8 displays the calculated excitation spectrum of the various BCN models over the range 0–7 eV. Results here were obtained via time-dependent DFT (TD-DFT) calculations, again, using the HSE06 exchange functional, implemented within the Amsterdam Density Functional program.^66^ A watermarked spectrum of the solar spectral irradiance as a function of energy is included to guide the reader toward where the material is deemed “photoactive” in a solar sense. Data for this spectrum was taken from an online database on the standard solar spectrum.^67^

For structures BN, BN-C_2_, and BN-C_6_, we observe the absorption edge outside of the solar spectral range and so cannot be considered as photoactive catalysts. With BN-C_24_, its absorption peak lies on the just on the edge of the solar spectrum at around 3.2 eV. This means that, although some of the higher energy photons might excite the material, their intensity is low (solar irradiance is 1/2 its peak value) reducing its efficiency as a photocatalyst. BN-C_54_ has its strongest excitation located in the middle of the solar spectrum, at 2.4 eV, making it a good candidate for photocatalysis. At this energy, the absorbed photon will possess enough energy to drive the catalytic reaction for the HER/OER process (1.23 V). The BN-C_96_ system also offers a material with good photoactive properties with two absorption peaks located within the solar spectral range, a major peak at 1.9 eV and a smaller one at 3 eV. Again, the minimum energy of the photon harvested by the material is greater than the energy required to drive the splitting of H_2_O at 1.23 V. Thus, out of the three viable materials, BN-C_54_ and BN-C_96_ both show good optical response in the solar spectrum and, therefore, could be potential candidates for environmentally friendly, photocatalytic H_2_O splitting.

## Conclusions

The present study systematically investigated the optoelectronic properties of seven h-BCN systems, starting from intrinsic h-BN and increasing C domain until graphene flake is realized, by means of DFT computations. Our results indicated that structures below 5 at. % C had high formation energies but the stability of structures with ≥10 at. % C showed relatively good stability with respect to h-BN. In particular, the BN-C_24_ system was calculated to be more stable than intrinsic BN. Electronic band gaps were found to decrease with increasing C content, from 5.94 eV (h-BN) to 4.67 eV at 1 at. % C down to as low as 1.61 eV at 44 at. % C. We also report three configurations providing appropriate band gap energies (1.6–3.0 eV) and band edge potentials appropriate for H_2_O oxidation and H_2_ and CO_2_ reduction. We identify structures with carbon content ≥10% as being potentially viable catalytic materials for future study under conditions near room temperature and near neutral pH, as band edges exhibited good alignment and small offset voltages with the reduction and oxidation potentials. We further investigated their light absorption properties to assess their efficacy as photocatalysts and found two morphologies that showed good optical response in the solar spectrum. Our results reveal that the optimal phase-separated h-BCN structures, BN-C_54_ and BN-C_96_, can serve as efficient photocatalysts for overall water splitting without the need for cocatalysts and propose them as candidates for further study. To comprehensively investigate their photocatalytic activity, future work will focus on the mechanism of both water oxidation and hydrogen reduction half reactions on these structures, looking at various reaction sites along the BN-C interface. By looking at the energies of adsorption, reaction, and desorption of H_2_O and its products, the efficacy of these systems as photocatalysts will be further evaluated.

## Supporting Information Available

Partial density of states–orbital contributions (Figure S1); XC functional evaluation (Table S1) (PDF)

## Abbreviations

h-BCN: hexagonal borocarbonitride
HER: hydrogen evolution reaction
OER: oxygen evolution reaction
DFT: Density Functional Theory
HSE06: Heyd Scuzeria and Ernzerhof hybrid functional
E_g_: material band gap
STM: Scanning Tunneling Microscopy
HOMO: highest occupied molecular orbital
LUMO: lowest unoccupied molecular orbital
NHE: Normal Hydrogen Electrode

## References

[ref1] Rao C. N. R.; Chhetri M. Borocarbonitrides as Metal-Free Catalysts for the Hydrogen Evolution Reaction. Adv. Mater. 2019, 31 (13), 1803668 10.1002/adma.201803668. 30375670

[ref2] Wang S.; Zhang L.; Xia Z.; Roy A.; Chang D. W.; Baek J.-B.; Dai L. BCN Graphene as Efficient Metal-Free Electrocatalyst for the Oxygen Reduction Reaction. Angew. Chem., Int. Ed. 2012, 51 (17), 4209–4212. 10.1002/anie.201109257. 22431416

[ref3] Huang C.; Chen C.; Zhang M.; Lin L.; Ye X.; Lin S.; Antonietti M.; Wang X. Carbon-doped BN nanosheets for metal-free photoredox catalysis. Nat. Commun. 2015, 6 (1), 7698 10.1038/ncomms8698. 26159752 PMC4510690

[ref4] Yuan T.; Zheng M.; Antonietti M.; Wang X. Ceramic boron carbonitrides for unlocking organic halides with visible light. Chem. Sci. 2021, 12 (18), 6323–6332. 10.1039/D1SC01028J. 34084430 PMC8115245

[ref5] Zheng M.; Shi J.; Yuan T.; Wang X. Metal-Free Dehydrogenation of N-Heterocycles by Ternary h-BCN Nanosheets with Visible Light. Angew. Chem., Int. Ed. 2018, 57 (19), 5487–5491. 10.1002/anie.201800319. 29473268

[ref6] Chang B.; et al. Metal-free boron carbonitride with tunable boron Lewis acid sites for enhanced nitrogen electroreduction to ammonia. Appl. Catal., B 2021, 283, 119622 10.1016/j.apcatb.2020.119622.

[ref7] Ahsan M. A.; He T.; Eid K.; Abdullah A. M.; Curry M. L.; Du A.; Santiago A. R. P.; Echegoyen L.; Noveron J. C. Tuning the Intermolecular Electron Transfer of Low-Dimensional and Metal-Free BCN/C60 Electrocatalysts via Interfacial Defects for Efficient Hydrogen and Oxygen Electrochemistry. J. Am. Chem. Soc. 2021, 143 (2), 1203–1215. 10.1021/jacs.0c12386. 33401899

[ref8] Jiménez-Arévalo N.; Leardini F.; Ferrer I. J.; Ares J. R.; Sánchez C.; Saad Abdelnabi M. M.; Betti M. G.; Mariani C. Ultrathin Transparent B–C–N Layers Grown on Titanium Substrates with Excellent Electrocatalytic Activity for the Oxygen Evolution Reaction. ACS Appl. Energy Mater. 2020, 3 (2), 1922–1932. 10.1021/acsaem.9b02339.

[ref9] Joshi P.; Yadav R.; Hara M.; Inoue T.; Motoyama Y.; Yoshimura M. Contribution of B,N-co-doped reduced graphene oxide as a catalyst support to the activity of iridium oxide for oxygen evolution reaction. J. Mater. Chem. A 2021, 9 (14), 9066–9080. 10.1039/D1TA00158B.

[ref10] Zhou M.; Wang S.; Yang P.; Huang C.; Wang X. Boron Carbon Nitride Semiconductors Decorated with CdS Nanoparticles for Photocatalytic Reduction of CO2. ACS Catal. 2018, 8 (6), 4928–4936. 10.1021/acscatal.8b00104.

[ref11] Tabassum H.; Zou R.; Mahmood A.; Liang Z.; Guo S. A catalyst-free synthesis of B, N co-doped graphene nanostructures with tunable dimensions as highly efficient metal free dual electrocatalysts. J. Mater. Chem. A 2016, 4 (42), 16469–16475. 10.1039/C6TA07214C.

[ref12] Angizi S.; Akbar M. A.; Darestani-Farahani M.; Kruse P. Review*—*Two-Dimensional Boron Carbon Nitride: A Comprehensive Review. ECS J. Solid State Sci. Technol. 2020, 9 (8), 083004 10.1149/2162-8777/abb8ef.

[ref13] Nehate S. D.; Saikumar A. K.; Prakash A.; Sundaram K. B. A review of boron carbon nitride thin films and progress in nanomaterials. Mater. Today Adv. 2020, 8, 100106 10.1016/j.mtadv.2020.100106.

[ref14] Guo F.; Yang P.; Pan Z.; Cao X.-N.; Xie Z.; Wang X. Carbon-Doped BN Nanosheets for the Oxidative Dehydrogenation of Ethylbenzene. Angew. Chem., Int. Ed. 2017, 56 (28), 8231–8235. 10.1002/anie.201703789. 28514048

[ref15] Wang S.; Ma F.; Jiang H.; Shao Y.; Wu Y.; Hao X. Band gap-Tunable Porous Borocarbonitride Nanosheets for High Energy-Density Supercapacitors. ACS Appl. Mater. Interfaces 2018, 10 (23), 19588–19597. 10.1021/acsami.8b02317. 29775049

[ref16] Koós A. A.; et al. Effects of temperature and ammonia flow rate on the chemical vapour deposition growth of nitrogen-doped graphene. Phys. Chem. Chem. Phys. 2014, 16 (36), 19446–19452. 10.1039/C4CP02132K. 25103112

[ref17] Qiao M.; Meysami S. S.; Ferrero G. A.; Xie F.; Meng H.; Grobert N.; Titirici M.-M. Low-Cost Chitosan-Derived N-Doped Carbons Boost Electrocatalytic Activity of Multiwall Carbon Nanotubes. Adv. Funct. Mater. 2018, 28 (16), 1707284 10.1002/adfm.201707284.

[ref18] Tay R. Y.; Wang X.; Tsang S. H.; Loh G. C.; Singh R. S.; Li H.; Mallick G.; Tong Teo E. H. A systematic study of the atmospheric pressure growth of large-area hexagonal crystalline boron nitride film. J. Mater. Chem. C 2014, 2 (9), 1650–1657. 10.1039/c3tc32011a.

[ref19] Han Z. J.; et al. High-frequency supercapacitors based on doped carbon nanostructures. Carbon 2018, 126, 305–312. 10.1016/j.carbon.2017.10.014.

[ref20] Wu Z.-S.; Ren W.; Xu L.; Li F.; Cheng H.-M. Doped Graphene Sheets As Anode Materials with Superhigh Rate and Large Capacity for Lithium Ion Batteries. ACS Nano 2011, 5 (7), 5463–5471. 10.1021/nn2006249. 21696205

[ref21] Reddy A. L. M.; Srivastava A.; Gowda S. R.; Gullapalli H.; Dubey M.; Ajayan P. M. Synthesis Of Nitrogen-Doped Graphene Films For Lithium Battery Application. ACS Nano 2010, 4 (11), 6337–6342. 10.1021/nn101926g. 20931996

[ref22] Bi Y.-S.; Liu B.; Liu X.-Y.; Qin Y.; Zou B.-X. A h-BCN for Electrochemical Sensor of Dopamine and Uric Acid. J. Nanomater. 2020, 12, 27361–27367. 10.1155/2020/4604820.

[ref23] Shirodkar S. N.; Waghmare U. V.; Fisher T. S.; Grau-Crespo R. Engineering the electronic bandgaps and band edge positions in carbon-substituted 2D boron nitride: a first-principles investigation. Phys. Chem. Chem. Phys. 2015, 17 (20), 13547–13552. 10.1039/C5CP01680K. 25940395

[ref24] Wang Z.; Luo Z.; Li J.; Yang K.; Zhou G. 2D van der Waals heterostructures of graphitic BCN as direct Z-scheme photocatalysts for overall water splitting: the role of polar π-conjugated moieties. Phys. Chem. Chem. Phys. 2020, 22 (41), 23735–23742. 10.1039/D0CP04219F. 33057521

[ref25] Mir S. H.; Yadav V. K.; Singh J. K. Boron–Carbon–Nitride Sheet as a Novel Surface for Biological Applications: Insights from Density Functional Theory. ACS Omega 2019, 4 (2), 3732–3738. 10.1021/acsomega.8b03454. 31459586 PMC6648852

[ref26] Thomas S.; Manju M. S.; Ajith K. M.; Lee S. U.; Asle Zaeem M. Strain-induced work function in h-BN and BCN monolayers. Phys. E 2020, 123, 114180 10.1016/j.physe.2020.114180.

[ref27] Karmakar S.; Dutta S. Strain-tuneable photocatalytic ability of BC6N monolayer: A first principle study. Comput. Mater. Sci. 2022, 202, 111002 10.1016/j.commatsci.2021.111002.

[ref28] Wang C.; Zhou X.; Li Y. Penta-BCN monolayer: a metal-free photocatalyst with a high carrier mobility for water splitting. Phys. Chem. Chem. Phys. 2022, 24 (43), 26863–26869. 10.1039/D2CP03311A. 36317519

[ref29] Nozaki H.; Itoh S. Structural stability of BC2N. J. Phys. Chem. Solids 1996, 57 (1), 41–49. 10.1016/0022-3697(95)00088-7.

[ref30] Thomas S.; Lee S. U. Atomistic insights into the anisotropic mechanical properties and role of ripples on the thermal expansion of h-BCN monolayers. RSC Adv. 2019, 9 (3), 1238–1246. 10.1039/C8RA08076C. 35518025 PMC9059568

[ref31] Blase X.; Charlier J. C.; De Vita A.; Car R. Theory of composite BxCyNz nanotube heterojunctions. Appl. Phys. Lett. 1997, 70 (2), 197–199. 10.1063/1.118354.

[ref32] Ci L.; et al. Atomic layers of hybridized boron nitride and graphene domains. Nat. Mater. 2010, 9 (5), 430–435. 10.1038/nmat2711. 20190771

[ref33] Wang Y.; Meng J.; Tian Y.; Chen Y.; Wang G.; Yin Z.; Jin P.; You J.; Wu J.; Zhang X.; et al. Deep Ultraviolet Photodetectors Based on Carbon-Doped Two-Dimensional Hexagonal Boron Nitride. ACS Appl. Mater. Interfaces 2020, 12 (24), 27361–27367. 10.1021/acsami.0c05850. 32449615

[ref34] Lu J.; Zhang X.; Liu X. F.; Zhang H.; Sum T. H.; Castro Neto A. H.; Loh K. P. Order–disorder transition in a two-dimensional boron–carbon–nitride alloy. Nat. Commun. 2013, 4 (1), 2681 10.1038/ncomms3681. 24157959

[ref35] Li M.; et al. Growth and Etching of Centimeter-Scale Self-Assembly Graphene–h-BN Super-Ordered Arrays: Implications for Integrated Electronic Devices. ACS Appl. Nano Mater. 2022, 5 (1), 774–781. 10.1021/acsanm.1c03516.

[ref36] Geng D.; et al. One-Pot Confined Epitaxial Growth of 2D Heterostructure Arrays. ACS Mater. Lett. 2021, 3 (2), 217–223. 10.1021/acsmaterialslett.0c00517.

[ref37] Perdew J. P.; Yue W. Accurate and simple density functional for the electronic exchange energy: Generalized gradient approximation. Phys. Rev. B 1986, 33 (12), 8800–8802. 10.1103/PhysRevB.33.8800. 9938293

[ref38] Becke A. D. Density-functional exchange-energy approximation with correct asymptotic behavior. Phys. Rev. A 1988, 38 (6), 3098–3100. 10.1103/PhysRevA.38.3098. 9900728

[ref39] Perdew J. P.; Ruzsinszky A.; Csonka G. I.; Constantin L. A.; Sun J. Workhorse Semilocal Density Functional for Condensed Matter Physics and Quantum Chemistry. Phys. Rev. Lett. 2009, 103 (2), 026403 10.1103/PhysRevLett.103.026403. 19659225

[ref40] Perdew J. P.; Ruzsinszky A.; Csonka G. I.; Constantin L. A.; Sun J. Erratum: Workhorse Semilocal Density Functional for Condensed Matter Physics and Quantum Chemistry [Phys. Rev. Lett. 2009, 103 (17), 026403 10.1103/PhysRevLett.103.026403. 19659225

[ref41] Tran F.; Blaha P. Accurate Band Gaps of Semiconductors and Insulators with a Semilocal Exchange-Correlation Potential. Phys. Rev. Lett. 2009, 102 (22), 226401 10.1103/PhysRevLett.102.226401. 19658882

[ref42] Koller D.; Tran F.; Blaha P. Improving the modified Becke-Johnson exchange potential. Phys. Rev. B 2012, 85 (15), 155109 10.1103/PhysRevB.85.155109.

[ref43] Heyd J.; Scuseria G. E.; Ernzerhof M. Hybrid functionals based on a screened Coulomb potential. J. Chem. Phys. 2003, 118 (18), 8207–8215. 10.1063/1.1564060.

[ref44] Heyd J.; Scuseria G. E.; Ernzerhof M. Erratum: “Hybrid functionals based on a screened Coulomb potential” [J. Chem. Phys. 118, 8207 (2003)]. J. Chem. Phys. 2006, 124 (21), 219906 10.1063/1.2204597.

[ref45] Elias C.; Valvin P.; Pelini T.; Summerfield A.; Mellor C. J.; Cheng T. S.; Eaves L.; Foxon C. T.; Beton P. H.; Novikov S. V.; Gil B.; et al. Direct band-gap crossover in epitaxial monolayer boron nitride. Nat. Commun. 2019, 10 (1), 2639 10.1038/s41467-019-10610-5. 31201328 PMC6572751

[ref46] Shima K.; Cheng T. S.; Mellor C. J.; Beton P. H.; Elias C.; Valvin P.; Gil B.; Cassabois G.; Novikov S. V.; Chichibu S. F. Cathodoluminescence spectroscopy of monolayer hexagonal boron nitride. Sci. Rep. 2024, 14 (1), 169 10.1038/s41598-023-50502-9. 38167439 PMC10762211

[ref47] Kim D.-H.; Kim H.-S.; Song M. W.; Lee S.; Lee S. Y. Geometric and electronic structures of monolayer hexagonal boron nitride with multi-vacancy. Nano Convergence 2017, 4 (1), 13 10.1186/s40580-017-0107-0. 28616375 PMC5446557

[ref48] Asif Q. U. A.; Hussain A.; Nabi A.; Tayyab M.; Rafique H. M. Computational study of X-doped hexagonal boron nitride (h-BN): structural and electronic properties (X = P, S, O, F, Cl). J. Mol. Model. 2021, 27 (2), 31 10.1007/s00894-020-04659-z. 33415475

[ref49] Ren X.-Y.; Xia S.; Li X.-B.; Chen N.-K.; Wang X.-P.; Wang D.; Chen Z.-G.; Zhang S.; Sun H.-B. Non-phase-separated 2D B–C–N alloys via molecule-like carbon doping in 2D BN: atomic structures and optoelectronic properties. Phys. Chem. Chem. Phys. 2018, 20 (35), 23106–23111. 10.1039/C8CP03028F. 30168546

[ref50] Geim A. K.; Novoselov K. S. The rise of graphene. Nat. Mater. 2007, 6 (3), 183–191. 10.1038/nmat1849. 17330084

[ref51] Sofo J. O.; Chaudhari A. S.; Barber G. D. Graphane: A two-dimensional hydrocarbon. Phys. Rev. B 2007, 75 (15), 153401 10.1103/PhysRevB.75.153401.

[ref52] Xiang H.; Kan E.; Wei S.-H.; Whangbo M.-H.; Yang J. Narrow” Graphene Nanoribbons Made Easier by Partial Hydrogenation. Nano Lett 2009, 9 (12), 4025–4030. 10.1021/nl902198u. 19995081

[ref53] Hu W.; Lin L.; Yang C.; Yang J. Electronic structure and aromaticity of large-scale hexagonal graphene nanoflakes. J. Chem. Phys. 2014, 141 (21), 214704 10.1063/1.4902806. 25481158

[ref54] Lanzillo N. A.; Kharche N.; Nayak S. K. Substrate-induced Band Gap Renormalization in Semiconducting Carbon Nanotubes. Sci. Rep. 2014, 4 (1), 3609 10.1038/srep03609. 24402238 PMC3885876

[ref55] Trasatti S. The absolute electrode potential: an explanatory note (Recommendations 1986). Pure Appl. Chem. 1986, 58 (7), 955–966. 10.1351/pac198658070955.

[ref56] Sun Z.; Ma T.; Tao H.; Fan Q.; Han B. Fundamentals and Challenges of Electrochemical CO2 Reduction Using Two-Dimensional Materials. Chem. 2017, 3 (4), 560–587. 10.1016/j.chempr.2017.09.009.

[ref57] Hoffmann M. R.; Martin S. T.; Choi W.; Bahnemann D. W. Environmental Applications of Semiconductor Photocatalysis. Chem. Rev. 1995, 95 (1), 69–96. 10.1021/cr00033a004.

[ref58] Fischer M.; Caridad J. M.; Sajid A.; Ghaderzadeh S.; Ghorbani-Asl M.; Gammelgaard L.; Bøggild P.; Thygesen K. S.; Krasheninnikov A. V.; Xiao S.; Wubs M.; et al. Controlled generation of luminescent centers in hexagonal boron nitride by irradiation engineering. Sci. Adv. 2021, 7 (8), eabe7138 10.1126/sciadv.abe7138. 33597249 PMC7888958

[ref59] Liu H.; et al. Rational Control on Quantum Emitter Formation in Carbon-Doped Monolayer Hexagonal Boron Nitride. ACS Appl. Mater. Interfaces 2022, 14 (2), 3189–3198. 10.1021/acsami.1c21781. 34989551

[ref60] Mendelson N.; et al. Identifying carbon as the source of visible single-photon emission from hexagonal boron nitride. Nat. Mater. 2021, 20 (3), 321–328. 10.1038/s41563-020-00850-y. 33139892

[ref61] Nørskov J. K.; Rossmeisl J.; Logadottir A.; Lindqvist L.; Kitchin J. R.; Bligaard T.; Jónsson H. Origin of the Overpotential for Oxygen Reduction at a Fuel-Cell Cathode. J. Phys. Chem. B 2004, 108 (46), 17886–17892. 10.1021/jp047349j. 39682080

[ref62] Yu E. T.; McCaldin J. O.; McGill T. C. Band Offsets in Semiconductor Heterojunctions Solid State Phys. 1992, 46. 1–146. 10.1016/S0081-1947(08)60397-5.

[ref63] Zhou Z.; Pei Z.; Wei L.; Zhao S.; Jian X.; Chen Y. Electrocatalytic hydrogen evolution under neutral pH conditions: current understandings, recent advances, and future prospects. Energy Environ. Sci. 2020, 13 (10), 3185–3206. 10.1039/D0EE01856B.

[ref64] Sherrell P. C.; Palczynski P.; Sokolikova M. S.; Reale F.; Pesci F. M.; Och M.; Mattevi C. Large-Area CVD MoS2/WS2 Heterojunctions as a Photoelectrocatalyst for Salt-Water Oxidation. ACS Appl. Energy Mater. 2019, 2 (8), 5877–5882. 10.1021/acsaem.9b01008.

[ref65] Valeur B.; Berberan-Santos M. N.; Molecular Fluorescence: principles and Applications; Wiley, 2012.

[ref66] van Gisbergen S. J. A.; Snijders J. G.; Baerends E. J. Implementation of time-dependent density functional response equations. Comput. Phys. Commun. 1999, 118 (2), 119–138. 10.1016/S0010-4655(99)00187-3.

[ref67] Standard Solar Spectrum - ASTM G-173. accessed 21 October 2024; https://www.pveducation.org/pvcdrom/appendices/standard-solar-spectra.

