# Supplementary material for: Density Functional Theory Investigation of 2D Phase Separated Graphene/Hexagonal Boron Nitride Monolayers; Band Gap, Band Edge Positions, and Photo Activity
Source: J Phys Chem C Nanomater Interfaces. 2024 Nov 27;129(1):638–47. doi: 10.1021/acs.jpcc.4c06121 (PMC11726656; doi:10.1021/acs.jpcc.4c06121)
Supplement: Supplementary file 1 — jp4c06121_si_001.pdf [file jp4c06121_si_001.pdf]

Density Functional Theory Investigation of 2D Phase Separated Graphene / Hexagonal Boron Nitride Monolayers; Bandgap, Band Edge Positions and Photo-Activity

*Eoin M. O'Sullivan [1], Nicole Grobert [1], Marcel Swart [2,3]**

[1] Department of Materials, University of Oxford, OX1 3PH Oxford, U.K.

[2] Institute de Quimica Computacional i Catálisi, Universitat de Girona, 17003 Girona, Spain

[3] ICREA, 08010 Barcelona, Spain

*Corresponding Author's Email: marcel.swart@udg.edu

1. Partial Density of States

S and P orbital contributions to PDOS

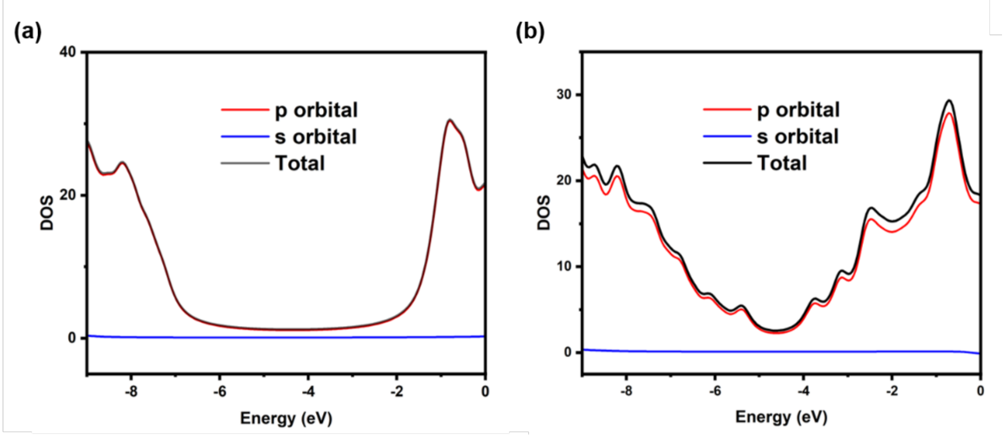

Figure S1. Partial density of states showing the s orbital and p orbital contributions in the (a) BN and (b) BN-C_{96} structure. In both undoped and highly C-doped structures, it is the p orbitals that contribute to the HOMO and LUMO states.

2. XC Functional Evaluation

The performance of various DFT functionals in determining the bandgap of intrinsic hBN were compared to choose the most suitable functional for assessing the hBCN systems.

Table S1. XC functional, calculated electronic band gap and % deviation from the fundamental bandgap of hBN. This is generally accepted as 5.95 eV for bulk hBN, however for monolayer hBN, it is reported to be 6.05 eV [1, 2]. As such, a bandgap of 6.05 eV is chosen as the experimental reference value here.

XC functional	Bandgap of BN (eV)	% dev.
BP86D3	4.53	25.1
revtpss	4.77	21.2
TB-MBJ	6.19	2.3
HSE06	5.94	1.8

Table S2. Band edge positions (relative to the vacuum level) for the hBN flake analysed in our study using the TB-MBJ and HSE06 functionals, and literature values for the band edge positions of hBN.

	HOMO / VBM (eV)	LUMO / CBM (eV)
TB-MBJ	-5.16	+1.03
HSE06	-7.23	-1.3
Shirodkar et al. [3] (DFT)	-6.6	-0.9
Zhang et al. [4] (Experimental)	-6.88	-1.18
Sangwan et al. [5] (Review)	-7.9	-2.0

For the hBN flake, results from the HSE06 XC functional align well with literature values for band edge positions of hBN, We obtained the highest occupied molecular orbital (HOMO) and lowest unoccupied molecular orbital (LUMO) energies at -7.23 eV and -1.3 eV, respectively, relative to the vacuum level. These values are in close agreement with both theoretical and experimental results previously reported for hBN. Previous calculations on a periodic model of a hBN monolayer (18 atoms) reported Valence Band Maximum (VBM) and Conduction Band Minimum (CBM) values of -6.6 eV and -0.9 eV, respectively [3]. Additionally, ultraviolet photoelectron spectroscopy (UPS) analysis of hBN nanosheets yielded experimental VBM and CBM values at -6.88 eV and -1.18 eV, respectively [4]. A review on electronic transport in two-dimensional materials provides similar hBN band edge positions, quoting VBM at -7.9 eV and CBM at -2.0 eV [5]. These literature values support the accuracy of our determination of band edges in the hBCN systems using the HSE06 functional, which is imperative for assessing the photocatalytic potentials in hydrogen evolution reaction (HER) and oxygen reduction reaction (ORR) applications.

REFERENCES

1. Elias, C., *et al.*, *Direct band-gap crossover in epitaxial monolayer boron nitride.* Nature Communications, 2019. **10**(1): p. 2639.
2. Shima, K., *et al.*, *Cathodoluminescence spectroscopy of monolayer hexagonal boron nitride.* Scientific Reports, 2024. **14**(1): p. 169.
3. Shirodkar, S.N., U.V. Waghmare, T.S. Fisher, and R. Grau-Crespo, *Engineering the electronic bandgaps and band edge positions in carbon-substituted 2D boron nitride: a first-principles investigation.* Physical Chemistry Chemical Physics, 2015. **17**(20): p. 13547-13552.
4. Zhang, Q., *et al.*, *Large-Area Self-Assembled Hexagonal Boron Nitride Nanosheet Films for Ultralow Dark Current Vacuum-Ultraviolet Photodetectors.* Advanced Functional Materials, 2024. **34**(28): p. 2315149.
5. Sangwan, V.K. and M.C. Hersam, *Electronic Transport in Two-Dimensional Materials.* Annual Review of Physical Chemistry, 2018. **69**(Volume 69, 2018): p. 299-325.
